# Vitreoretinal Interface Abnormalities in Patients With Retinal Vein Occlusion in a Tertiary Referral Center

**DOI:** 10.7759/cureus.66638

**Published:** 2024-08-11

**Authors:** Irini Chatziralli, Chrysa Agapitou, Eleni Dimitriou, Petros Kapsis, Dimitrios Kazantzis, Alexia Risi-Koziona, Georgios Theodossiadis, Panagiotis Theodossiadis

**Affiliations:** 1 Second Department of Ophthalmology, National and Kapodistrian University of Athens, Athens, GRC

**Keywords:** epiretinal membrane, vitreomacular traction, optical coherence tomography, vitreoretinal interface, retinal vein occlusion

## Abstract

Purpose: The purpose of this study is to investigate the prevalence of vitreoretinal interface (VRI) disorders in patients with retinal vein occlusion (RVO) and to evaluate the impact of VRI abnormalities on the treatment outcomes of macular edema secondary to RVO using intravitreal aflibercept.

Methods: Participants in this prospective study were consecutive patients with macular edema secondary to RVO, who received intravitreal aflibercept injections. At baseline, best-corrected visual acuity (BCVA) was assessed, and spectral domain-optical coherence tomography (SD-OCT) was performed to measure central subfield thickness (CST) and to evaluate the presence of VRI disorders, namely, vitreoretinal adhesion (VMA), vitreoretinal traction (VMT), epiretinal membrane (ERM), lamellar macular hole (LMH), and full-thickness macular hole (FTMH). The primary outcomes were the prevalence of various VRI disorders in patients with RVO and the impact of VRI disorders on BCVA and CST after aflibercept treatment in such patients.

Results: At baseline, 16.1% of patients had VMA, 3.2% VMT, 18.3% ERM, and 1.1% LMH. There were a statistically significant improvement in BCVA and a decrease in CST in RVO patients over time. There was no statistically significant difference regarding BCVA and CST at baseline and until month 24 after treatment between patients with VRI disorders and those without VRI disorders. However, the mean number of injections during the follow-up period was higher in the group with VRI disorders (9.4±2.1) compared to those without VRI disorders (8.1±0.7, p=0.0002).

Conclusions: The prevalence of VRI disorders in patients with RVO was 16.1% for VMA, 3.2% for VMT, 18.3% for ERM, and 1.1% for LMH. VRI disorders were not found to affect the anatomical and visual outcomes after intravitreal aflibercept treatment in patients with RVO, although more intravitreal injections were needed in patients with VRI disorders.

## Introduction

Retinal vein occlusion (RVO), either central (CRVO) or branch (BRVO), is the second most common retinal vascular disorder after diabetic retinopathy and is a frequent cause of visual impairment, especially due to macular edema or ischemia. It is estimated to affect about 16 million people worldwide, while in 2015 the global prevalence of RVO was found to be about 0.77% in people aged 30-89 [1-3].

Vitreoretinal interface (VRI) disorders, such as epiretinal membrane (ERM), vitreoretinal adhesion (VMA), vitreoretinal traction (VMT), lamellar macular hole (LMH), and full-thickness macular hole (FTMH), have gain special interest with the advent of spectral domain-optical coherence tomography (SD-OCT), allowing the more detailed and objective evaluation of VRI abnormalities [4]. Although the prevalence of VRI disorders has been reported in the general population in various cohort studies [5,6], the association between VRI and RVO has not been studied in detail.

Intravitreal anti-vascular endothelial growth factor (anti-VEGF) injections have been shown to be effective and safe for the treatment of macular edema secondary to RVO in large clinical trials [7]. Recent meta-analyses have shown that intravitreal anti-VEGF treatment provided an overall mean improvement in visual acuity of about 14 letters at 12 months in patients with CRVO or BRVO [8,9], in line with real-world data reporting about 12 letters improvement in 12 months [10,11]. Several SD-OCT biomarkers have been studied as potential predictors of treatment response in patients with RVO with variable results [12-16]. Although previous studies have examined the impact of VRI disorders on intravitreal anti-VEGF treatment outcomes in patients with neovascular age-related macular degeneration and diabetic macular edema [17-22], there are few reports regarding the treatment of RVO in association with VRI disorders [23-26].

In light of the above, the purpose of this study is to investigate the prevalence of VRI disorders in patients with RVO and to evaluate the impact of VRI abnormalities on the treatment outcomes of macular edema secondary to RVO using intravitreal anti-VEGF agents.

This article was previously presented as a meeting abstract at the Controversies in Ophthalmology Congress 2024 (March 15-16, 2024, Athens, Greece).

## Materials and methods

Participants in this prospective study were consecutive patients with macular edema secondary to RVO, who were recruited at the Second Department of Ophthalmology, National and Kapodistrian University of Athens, Athens, Greece, between September 1, 2019, and August 31, 2023. Diagnosis of RVO was made clinically on the basis of the presence of retinal hemorrhages, retinal vein dilatation, tortuosity, and flame-shaped and dot-blot hemorrhages while confirmed by retinal imaging. Patients with other retinal diseases than RVO, corneal disease, uveitis, glaucoma, dry eye disease, trauma, and any previous intraocular surgery during the last six months were excluded from the study. The study protocol adhered to the tenets of the Declaration of Helsinki and was approved by the Institutional Review Board of Attikon University Hospital (approval number: 699/2019). Written informed consent to participate in the study and for publication of data was obtained by all participants before enrollment to the study.

Demographic data of patients (age, gender) were recorded. At baseline, all participants underwent a complete ophthalmic examination, including best-corrected visual acuity (BCVA) measurement by means of Snellen charts (converted to logMAR scale for statistical analysis), slit-lamp examination, dilated fundoscopy, SD-OCT, and fundus fluorescein angiography (FFA) using Spectralis (Spectralis HRA+OCT, Heidelberg Engineering, Heidelberg, Germany). SD-OCT was obtained using a standard acquisition protocol; six radial scans 3 mm long were performed at equally spaced angular orientations centered on the foveola. The OCT volume scan was performed on a 20×20-degree cube, consisting of 49 horizontal B-scans with 20 averaged frames per B-scan centered over the fovea. The following SD-OCT variables were recorded at baseline: CST (μm) and the presence of VRI disorders, namely, VMA, VMT, ERM, LMH, and FTMH.

All patients were treated with intravitreal 2.0 mg/0.05 ml aflibercept injections, following a protocol of at least three monthly injections and thereafter pro re nata (PRN). Re-treatment was performed if BCVA decreased by at least 1 Snellen line and/or CST reduced ≤10% or was ≥320 μm. All participants were followed up every month for six months after treatment initiation and then every two months until 24 months after treatment initiation. At each follow-up visit, patients underwent BCVA measurement and SD-OCT, while FFA was performed at the physician's discretion.

The primary outcomes were the prevalence of various VRI disorders in patients with RVO and the impact of VRI disorders on BCVA and CST after aflibercept treatment in such patients.

Statistical analysis

For the description of patients' characteristics, descriptive statistics were calculated; mean±standard deviation (SD) was used for continuous variables, while relative frequencies and percentages for categorical variables were reported. All variables were tested for normal distribution with the Kolmogorov-Smirnov test. Comparisons between independent groups were performed, using the Mann-Whitney-Wilcoxon test or t-test, as well as with chi-squared test or Fisher's exact test. Comparison between baseline and different time-points of the follow-up regarding BCVA and CST was performed using paired t-tests or Wilcoxon signed-rank tests, as appropriate. Given that four comparisons were done (baseline vs. months 6, 12, 18, and 24), Bonferroni correction was adopted, and statistical significance was set to 0.05/4=0.0125. In all other cases, p-value <0.05 was considered statistically significant. Statistical analysis was performed using Stata Statistical Software: Release 13 (2013; StataCorp LLC, College Station, Texas, United States).

## Results

Participants in the study were 93 patients with RVO, 41 with CRVO (44.1%), and 52 with BRVO (55.9%), who were treated with intravitreal aflibercept injections. The demographic and clinical characteristics of the study sample are shown in Table 1. The mean age of patients was 70.7±10.3 years. Forty-nine patients were male (52.7%) and 44 female (47.3%). The mean time between the onset of the symptoms and the RVO diagnosis was 1.5±1.3 weeks. 

**Table 1:** Demographic and clinical characteristics of the study sample.

	Whole population (n=93)	Central retinal vein occlusion (n=41)	Branch retinal vein occlusion (n=52)
Age (years, mean±SD)	70.7±10.3	71.4±11.6	70.4±9.4
Gender (n, %)			
Male	49 (52.7%)	29 (70.7%)	20 (38.5%)
Female	44 (47.3%)	12 (29.3%)	32 (61.5%)
Lens status (n, %)			
Phakic	22 (23.7%)	12 (29.3%)	10 (19.2%)
Pseudophakic	71 (76.3%)	29 (70.7%)	42 (80.8%)
Retinal vein occlusion duration (weeks, mean±SD)	1.5±1.3	1.1±1.0	1.7±1.4
Best-corrected visual acuity (logMAR, mean±SD)	0.69±0.09	0.82±0.11	0.54±0.08
Central subfield thickness (μm, mean±SD)	486.2±109.7	502.6±111.3	399.9±73.1
Vitreomacular adhesion (n, %)	15 (16.1%)	12 (29.3%)	3 (5.8%)
Vitreomacular traction (n, %)	3 (3.2%)	2 (4.9%)	1 (1.9%)
Epiretinal membrane (n, %)	17 (18.3%)	13 (31.7%)	4 (7.7%)
Lamellar macular hole (n, %)	1 (1.1%)	0 (0%)	1 (1.9%)
Full-thickness macular hole (n, %)	0 (0%)	0 (0%)	0 (0%)

At baseline, 15 out of 93 patients (16.1%) presented with VMA, three patients (3.2%) VMT, 17 patients (18.3%) ERM, and one patient (1.1%) LMH. In a subgroup analysis, the prevalence of VRI disorders was significantly higher in patients with CRVO than in those with BRVO (p<0.001 for VMA, VMT, and ERM, chi-squared test), while LMH occurrence did not differ between the two groups (p=0.372, chi-squared test).

**Figure 1:**
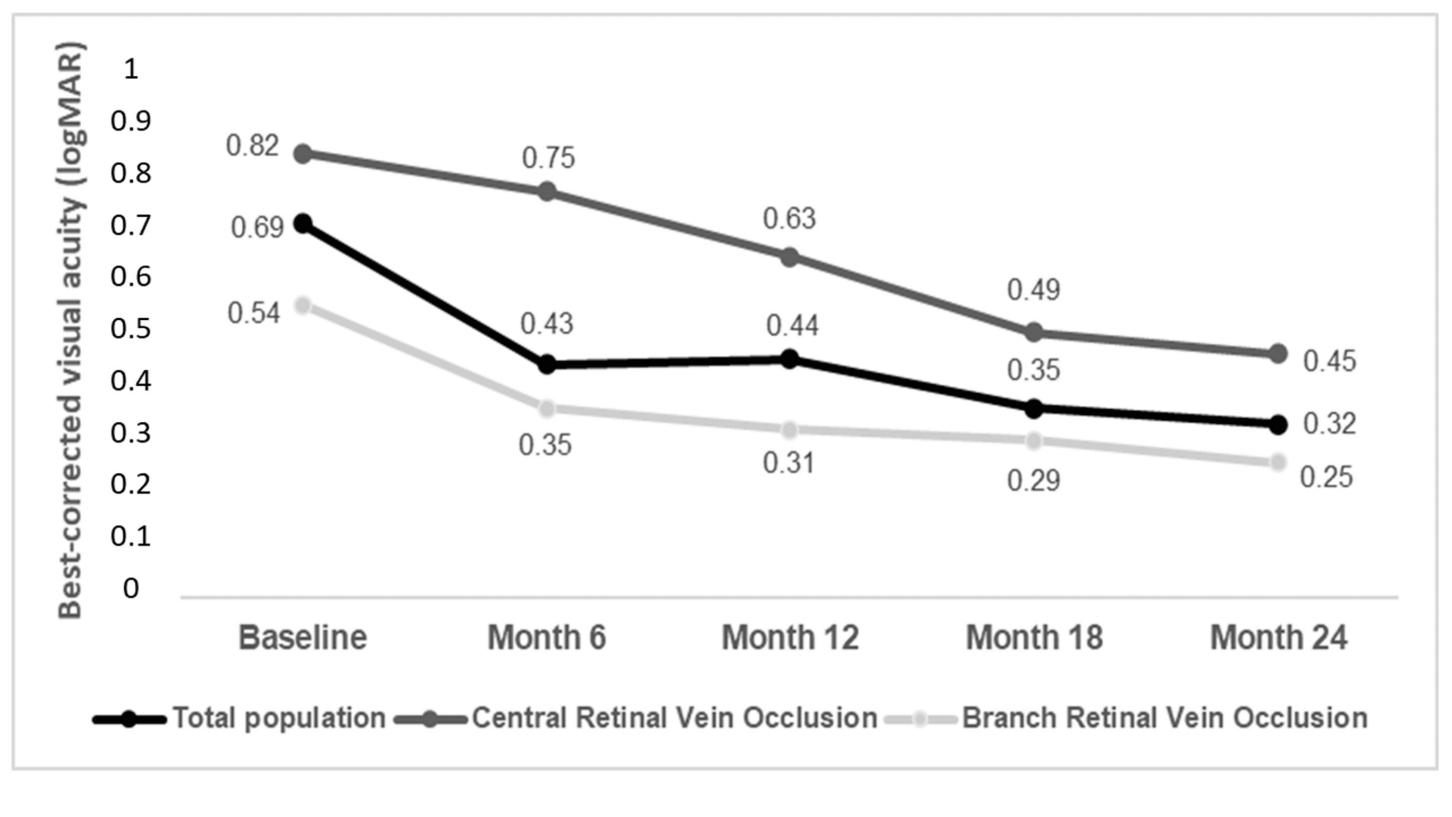
Evolution of best-corrected visual acuity over time.

The mean baseline BCVA was 0.69±0.09 logMAR. There was a statistically significant improvement in BCVA at all time-points of the follow-up in both CRVO and BRVO patients (p<0.001 for all comparisons), as it is depicted in Figure 1.

**Figure 2:**
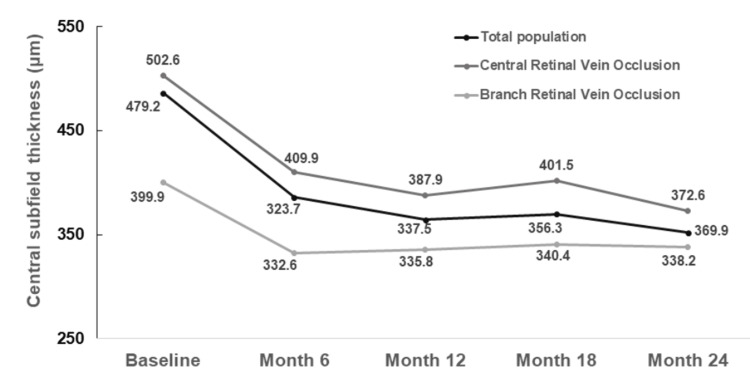
Evolution of central subfield thickness over time.

At baseline, the mean CST was 486.2±109.7 μm. There was a statistically significant decrease in CST at all time-points of the follow-up (p<0.001 for all comparisons), as it is shown in Figure 2. In a subgroup analysis, the CST significantly decreased in both CRVO and BRVO at all time-points of the follow-up (p<0.001 for all comparisons for CRVO and BRVO).

Table 2 shows the comparison between patients with VRI disorders and those without regarding BCVA and CST. There was no statistically significant difference between the two groups at all time-points of the follow-up, before or after intravitreal anti-VEGF treatment until month 24. However, the mean number of injections during the follow-up period was 9.4±2.1 in patients with VRI disorders and was significantly greater compared to those without VRI disorders (8.1±0.7, p=0.0002).

**Table 2:** Best-corrected visual acuity and central subfield thickness in patients with or without vitreoretinal interface disorders.

	Vitreoretinal interface disorders (n=38)	No vitreoretinal interface disorders (n=55)	P-value
Best-corrected visual acuity (logMAR, mean±SD)			
Baseline	0.71±0.09	0.69±0.10	0.326
Month 6	0.43±0.09	0.42±0.08	0.575
Month 12	0.44±0.06	0.43±0.09	0.551
Month 18	0.35±0.08	0.35±0.09	0.999
Month 24	0.32±0.08	0.31±0.10	0.609
Central subfield thickness (μm, mean±SD)			
Baseline	493.5±101.1	484.2±103.3	0.668
Month 6	389.6±85.2	384.4±81.3	0.767
Month 12	368.5±71.3	361.5±79.4	0.664
Month 18	370.9±70.7	367.2±70.9	0.805
Month 24	360.6±72.8	354.1±73.7	0.675

## Discussion

The principal message of our study is that the prevalence of the main VRI disorders in patients with RVO was 16.1% for VMA, 3.2% for VMT, 18.3% for ERM, and 1.1% for LMH. Of note, VRI disorders were more prevalent in patients with CRVO than in those with BRVO. In addition, intravitreal aflibercept was found to be effective in patients with RVO, either with or without VRI disorders. However, patients with VRI disorders needed more injections than those without VRI disorders.

Regarding the prevalence of VRI abnormalities in patients with RVO, Maggio et al. showed that VMA was present in 44.1% of patients with macular edema secondary to RVO, who were eligible for intravitreal injections, although the percentage did not differ significantly compared to controls (50.7%) [24]. Similar results were found by Terao et al., who reported that 47 out of 107 eyes (43.9%) with BRVO had VMA at baseline, before any intervention [25]. In our study, the prevalence of VRI disorders was significantly lower than in previous studies regarding RVO, which can be attributed to the fact that we included early-diagnosed patients with a mean duration of RVO of 1.5±1.3 weeks. In the Beaver Dam Eye Study, ERM was found in 34.1% of the general population, VMT in 1.6%, and LMH in 3.6%, prevalence which increased with age [5].

As far as the impact of VRI abnormalities on treatment response to intravitreal anti-VEGF agents in patients with macular edema secondary to RVO is concerned, the results of previous studies were controversial. Maggio et al. have demonstrated that VMA resulted in a more intensive treatment regimen, although it did not affect visual and anatomical outcomes in patients with RVO [24], in line with Hamam et al., who reported that ERM had no influence on bevacizumab efficacy in patients with CRVO-related macular edema during 12 months of treatment [27]. Similarly, Singh et al. showed no effect of VRI on treatment outcomes in RVO after six months of treatment [23]. On the other hand, Terao et al. reported superior anatomical and functional outcomes in patients with BRVO treated with bevacizumab in eyes with VMA [25]. Our study concluded that VRI abnormalities did not affect BCVA and CST after intravitreal aflibercept treatment, but patients with VRI disorders needed more injections than those without VRI disorders.

Potential limitations of the study pertain to the relatively small study sample. In addition, the study lacks a control group, to compare the prevalence of VRI disorders.

## Conclusions

Our study examined VRI abnormalities in patients with RVO, reporting lower prevalence of VRI disorders than previously described. Moreover, we found that VRI abnormalities did not affect the efficacy of intravitreal aflibercept in patients with RVO, providing a significant improvement in BCVA and a decrease in CST. However, more injections are needed in patients with VRI disorders. Further prospective studies with a large sample size may be useful to scrutinize our findings.
